# Effect of Amorphous TiO_2_ Nanoparticles on the Crystalline Structure and Functional Properties of P(VDF-TFE) Nanocomposites

**DOI:** 10.3390/ijms27188018

**Published:** 2026-09-09

**Authors:** Andrey A. Vodyashkin, Evgenia L. Buryanskaya, Polina M. Tyubaeva, Dmitriy S. Ryzhenko, Mstislav O. Makeev

**Affiliations:** 1 Bauman Moscow State Technical University, 2-я Baumanskaya St., 105005 Moscow, Russia; 2 Academic Department of Technology and Chemistry of Innovative Materials, Plekhanov University of Economics, 117997 Moscow, Russia; 3 Department of Biological and Chemical Physics of Polymers, Emanuel Institute of Biochemical Physics, Russian Academy of Sciences, 4 Kosygina Street, 119334 Moscow, Russia

**Keywords:** titanium dioxide nanoparticles, polyvinylidene fluoride, composite polymer materials, piezoelectrics, flexible electronics

## Abstract

In this study, a method for introducing titanium dioxide nanoparticles (TiO_2_NPs) into the polymer matrix of a ferroelectric copolymer of vinylidene fluoride with tetrafluoroethylene P(VDF-TFE) is proposed and optimized. A comprehensive analysis showed that TiO_2_NPs content has a significant effect on the structure formation processes in the polymer matrix, the degree of crystallinity, phase composition, and surface morphology of the composites. By optimizing the amount of doped nanoparticles, it is possible to increase the electrical strength and permittivity of the material, as well as enhance the piezoelectric response compared to a film without TiO_2_NPs. The introduction of TiO_2_NPs into the P(VDF/TFE) polymer matrix promotes efficient polarization of the composite film without preliminary high-temperature orientational drawing. The approaches presented in this study can simplify process operations in the manufacture of flexible sensors, wearable electronics, and other devices that require a combination of piezoelectric activity and high electrical strength.

## 1. Introduction

Polyvinylidene fluoride (PVDF) is a promising material for use in electrical devices and sensors. PVDF and its copolymers have proven themselves to be among the most promising materials for such applications due to their excellent flexibility, mechanical strength, and piezoelectric properties [1,2,3,4]. See the end of the document for further details on references.

One of the most rapidly developing areas is the creation of autonomous wearable systems for monitoring various parameters, including physiological ones [5,6]. Piezoelectric materials, including PVDF-based copolymers, are capable of converting the mechanical energy of human movements (walking, breathing, and pulsations) into an electrical signal, which makes it possible to create energy-independent devices [7]. Such devices are used in systems for continuous monitoring of heart rate, pressure, and temperature, as well as in smart textiles [8]. Integration with artificial intelligence and soft robotics paves the way to next-generation adaptive healthcare systems [9]. The electroactive properties of PVDF and PVDF-based copolymers are directly determined by their phase composition. High piezo- and pyroelectric activity is characteristic exclusively of polar β- and γ-phases [10,11]. Traditionally, the proportion of electroactive phases is increased by mechanical drawing or polarization in a strong field, which limits the design of products and requires complex processing equipment [12,13,14,15]. In recent years, the in-situ nucleation approach has been actively developed, where the introduction of nanoparticles serves as a trigger for the selective crystallization of the β-phase (or another necessary one) directly during film formation [16,17].

The introduction of nano- and micro-materials into the polymer matrix is a promising method for tuning the phase composition and electrophysical properties of PVDF and PVDF-based copolymers. Currently, various nanoparticles are successfully doped into the PVDF matrix [18]. They allow for optimization of crystallinity and phase composition and can also further functionalize the film [19,20].

Titanium dioxide nanoparticles (TiO_2_NPs) are safe, environmentally friendly, and biocompatible materials widely used in the paint and varnish, textile, pharmaceutical, and other industries [21,22,23]. Titanium dioxide nanoparticles can be obtained in a wide range of size characteristics and can be synthesized with specified crystallinity [24]. By changing the main characteristics of titanium dioxide nanoparticles (size, shape, hydrodynamic radius, crystallinity, phase composition, etc.), it is possible to control the final properties of the composite film [25,26,27].

Crystallinity is a key parameter for various materials. The crystallinity of titanium dioxide nanoparticles determines their electronic, optical, photocatalytic and mechanical properties, affecting the concentration of structural defects, charge-carrier transport, and chemical stability [28,29]. Increasing crystallinity usually improves conductivity, photocatalytic activity, and long-term stability of the material but may be accompanied by a decrease in specific surface area due to crystallite growth [30,31]. Titanium dioxide nanoparticles are capable of forming intermolecular interactions with the functional groups of PVDF, which can significantly alter its phase composition [32]. Furthermore, it has been shown that crystalline TiO_2_ nanoparticles can act as additional heterogeneous nucleation centers, facilitating the formation and growth of crystalline PVDF phases [33]. Together with the intrinsic electrophysical properties of crystalline polymorphs of TiO_2_, primarily anatase and rutile, these effects can increase electrical strength, reduce dielectric loss, and improve other functional properties of the polymer composite. However, despite the significant amount of research devoted to crystalline modifications of TiO_2_, the influence of amorphous TiO_2_ nanoparticles on the structure, properties, and electrophysical characteristics of PVDF-based films remains virtually unexplored to date.

In this study, a 94/6 copolymer of vinylidene fluoride and tetrafluoroethylene (P(VDF/TFE)) was chosen as the polymer matrix. The chosen copolymer, unlike the PVDF homopolymer, is more processable, dissolves in a larger number of solvents, and requires lower processing temperatures. It was previously shown [34] that P(VDF/TFE) copolymer films are not inferior to PVDF in terms of piezoelectric response. However, to achieve high values of piezoelectric response, preliminary orientational drawing of the films is necessary.

In our work, we propose a method for producing P(VDF/TFE)/TiO_2_NPs composite films with varying nanoparticle contents. We thoroughly examine the effect of doped nanoparticle concentration on the surface morphology, physicochemical, and electrical properties of P(VDF/TFE)/TiO_2_NPs.

## 2. Results and Discussion

### 2.1. Characterization of Titanium Dioxide Nanoparticles

**Figure 1:**
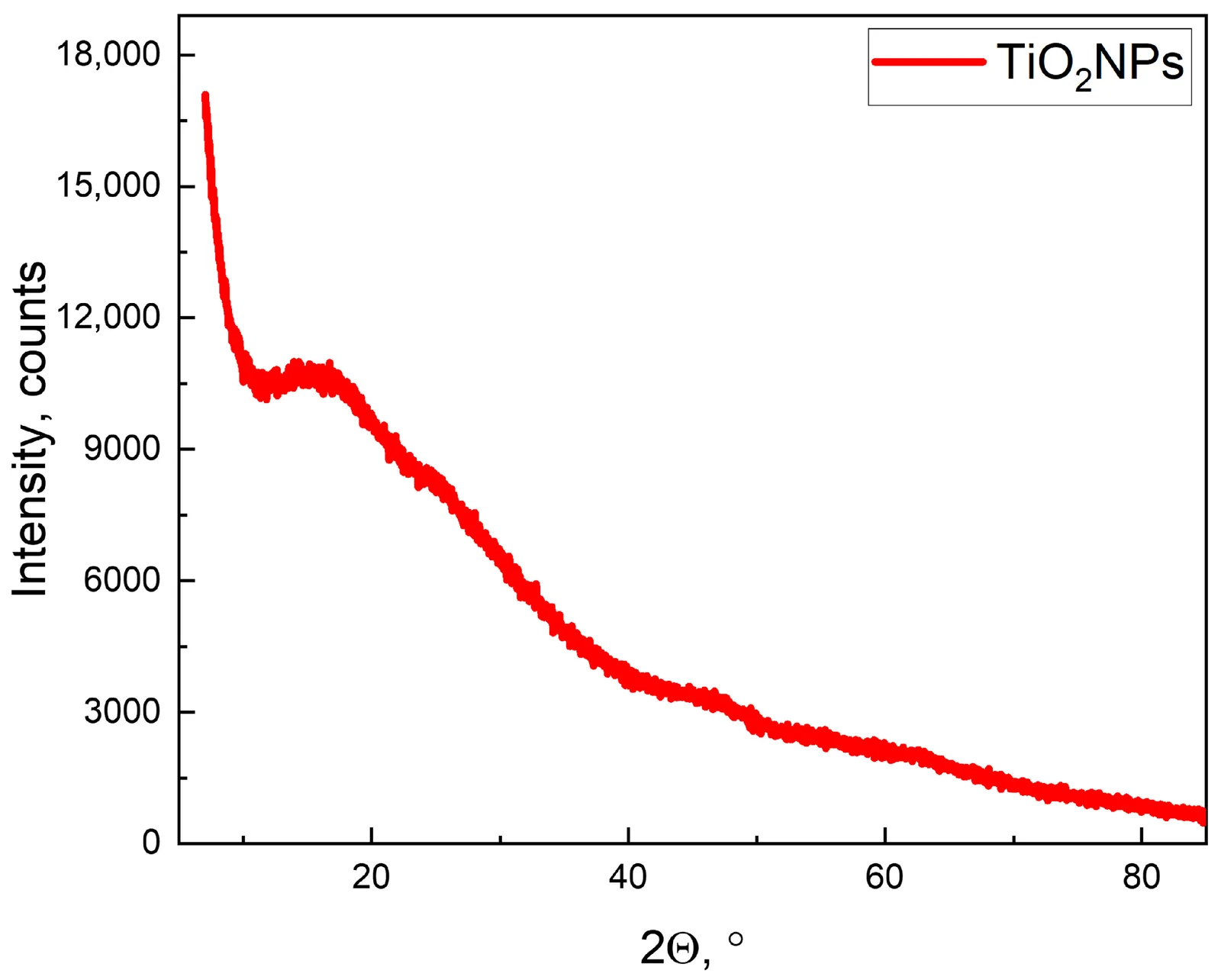
X-ray structural analysis of titanium dioxide nanoparticles.

The synthesis of titanium dioxide nanoparticles yielded a white powder, which agglomerated during drying. X-ray diffraction (XRD) confirmed the amorphous structure of the titanium dioxide nanoparticles (Figure 1).

**Figure 2:**
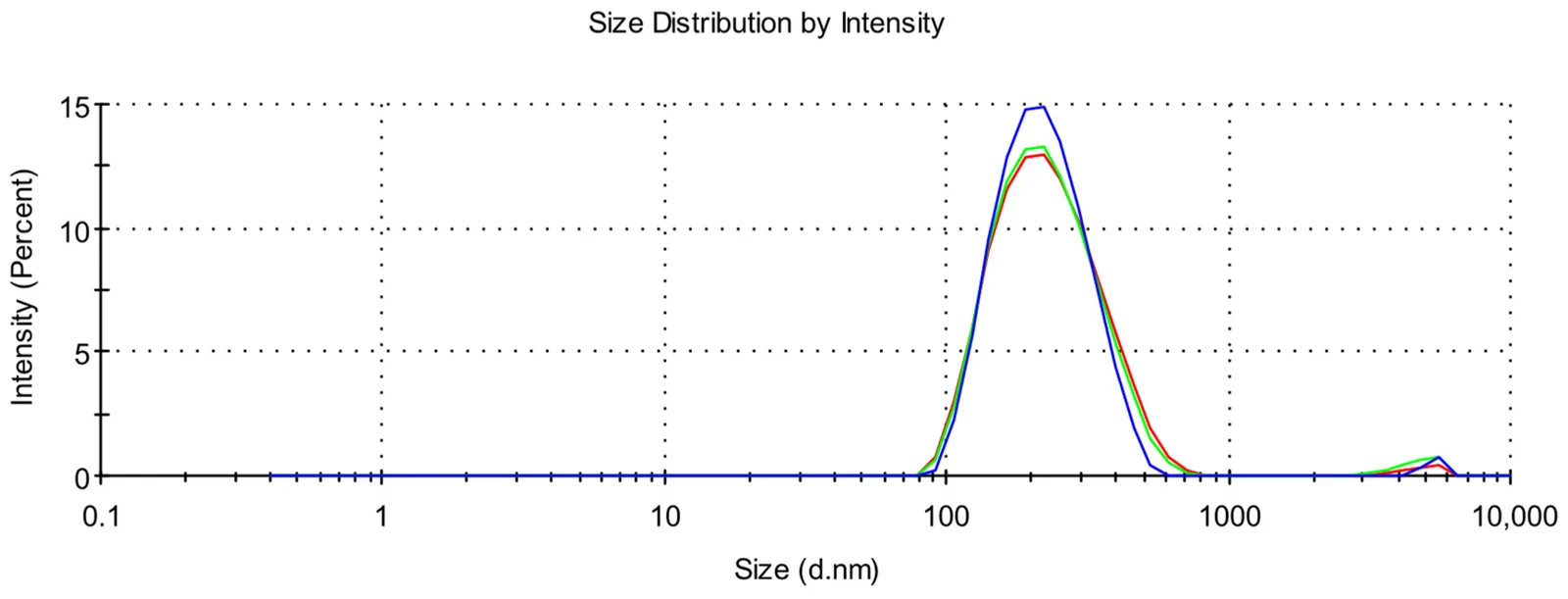
Hydrodynamic radius of titanium dioxide nanoparticles according to DLS data.

Dynamic light scattering (DLS) was used to characterize the nanoparticle sizes. Figure 2 shows the size distribution of titanium dioxide nanoparticles. The average hydrodynamic particle size was 215.7 (±82) nm (Table 1). A polydispersity index (PDI) of 0.187 indicates a relatively narrow particle size distribution and low polydispersity.

The high reproducibility of the results is confirmed by the fact that the standard deviation (of the average hydrodynamic diameter) between measurement series did not exceed 2 nm, indicating the stability of the studied dispersed system. The zeta potential of the aqueous sol of titanium dioxide nanoparticles was −25 mV. These values ensure sufficient electrostatic repulsion between particles and prevent aggregation, confirming the electrostatic mechanism of stabilization of the colloidal system.

### 2.2. FTIR Analysis of Composite Films P(VDF/TFE)/TiO_2_NPs

**Figure 3:**
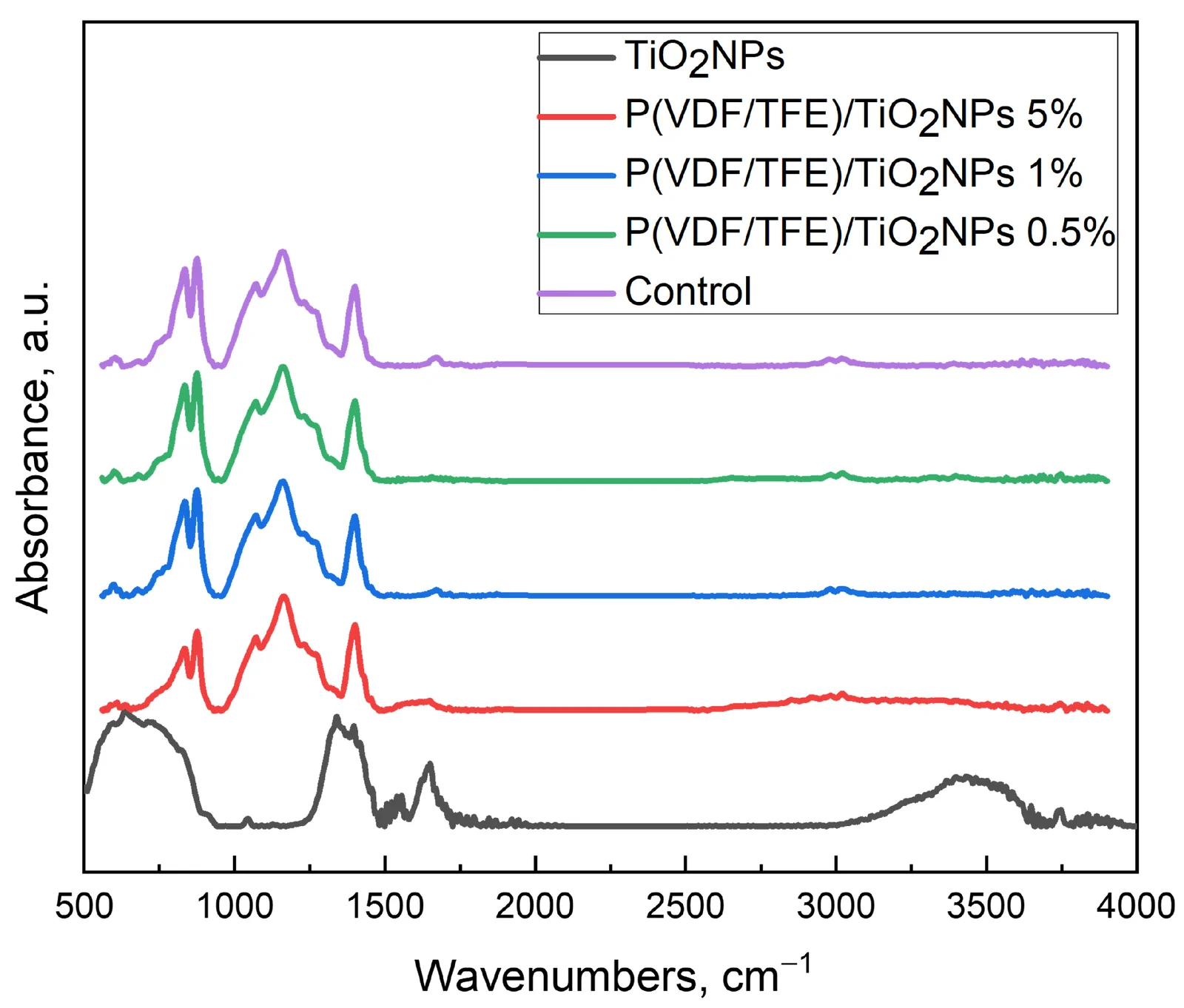
FTIR spectroscopy of P(VDF/TFE)/TiO_2_NPs composite polymer films.

Figure 3 shows FTIR spectroscopy data for composite films with different c contents. The spectra of P(VDF/TFE)/TiO_2_NPs 5% contain a diffuse band at 1600–1700 cm^−1^. It can be associated with crystalline bound water, which was incorporated into the nanoparticles during synthesis and drying. In samples without nanoparticles and with a content of 5 and 1%, the band at 1670 cm^−1^ indicates residual amounts of DMF. By selecting characteristic peaks (peaks influenced by only one phase) for specific PVDF phases, it is possible to analyze the influence of dopants on the phase composition of the composite material in accordance with [35]. As can be seen from Table 2, different concentrations of nanoparticles do not affect the total content of the electroactive phase (β- and γ-phases). According to the literature, the introduction of dopants into the polymer matrix should increase the β-phase content. However, as shown in Table 2, the nanoparticle concentration does not significantly affect the β-phase content. The addition of even 0.5% titanium dioxide nanoparticles leads to a significant change in phase composition and an increase in the electroactive phase within the system. However, a further increase in the nanoparticle content does not result in an increase in the β-phase. For the reference film, the β-phase content was 37%. After adding 0.5% TiO_2_NPs, the β-phase content increased to 49% but remained virtually unchanged (50%) with further increases in dopant concentration.

### 2.3. DSC Analysis of Composite Films P(VDF/TFE)/TiO_2_NPs

**Figure 4:**
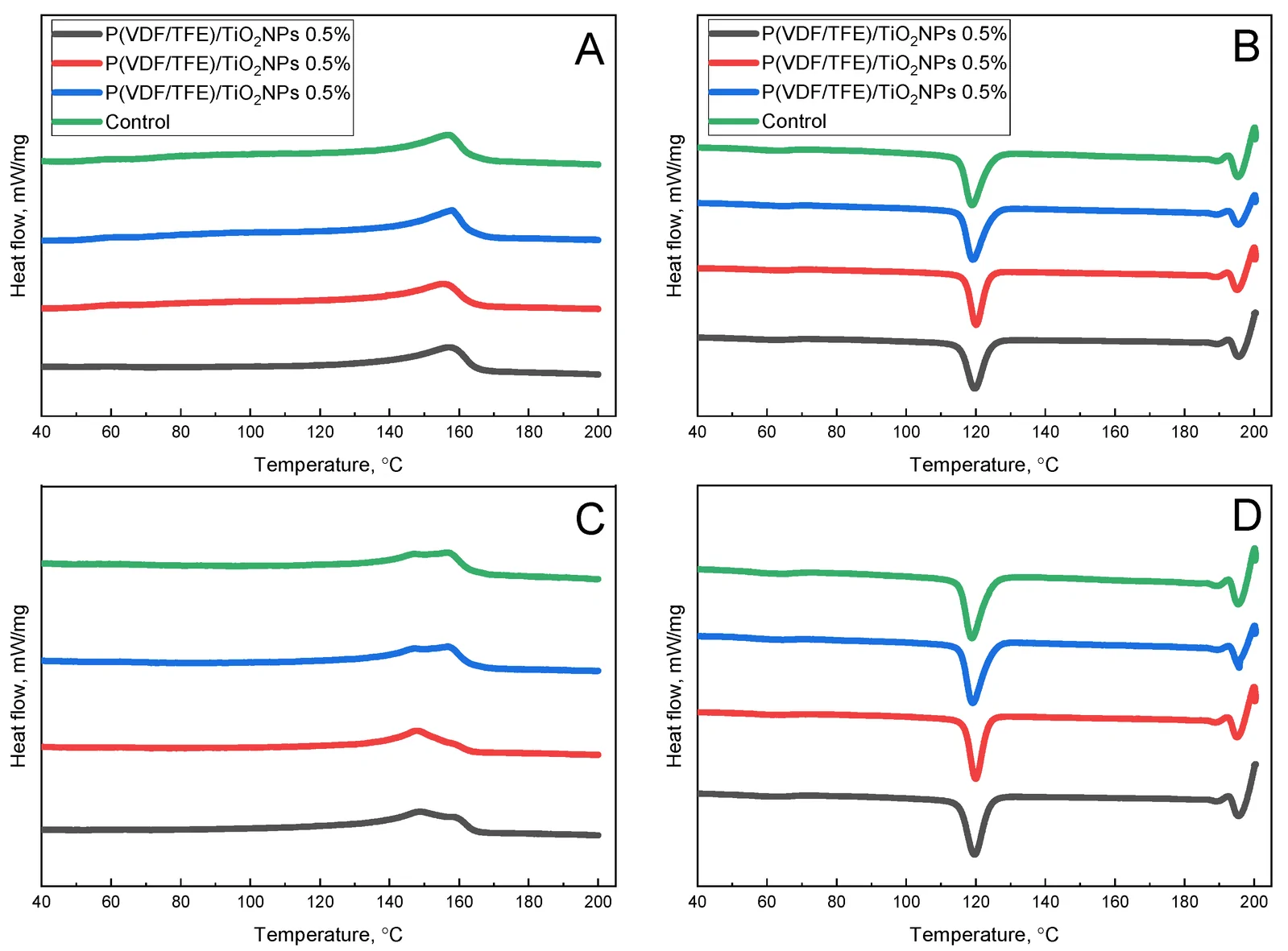
Data for DSC of P(VDF/TFE)/TiO_2_NPs composite materials for (**A**) 1 heating (**B**) 1 cooling (**C**) 2 heating (**D**) 2 cooling.

The DSC curves of composite films P(VDF/TFE)/TiO_2_NPs are shown in Figure 4. As can be observed from the DSC curves, the behavior of all samples during the first cycle of melting-crystallization is similar. All DSC curves during the first heating process (Figure 4A) are characterized by the onset of melting at approximately 140 °C, a broad melting peak in the range of 155–158 °C, and the completion of phase transition at around 165 °C. The first crystallization process displays a similar behavior (Figure 4B), with the onset of crystallization at approximately 130–127 °C and rapid crystallization in the range of 110 °C. The behavior of the samples during the second cycle of melting-crystallization has a similar nature of complex changes (Figure 4C). All samples have differentiated melting peak characterized by the onset of melting at approximately 135 °C and the completion of the phase transition around 165 °C. The samples exhibit a first melting maximum at 146–148 °C followed by a second melting maximum in the range of 156–159 °C. Nevertheless, it should be emphasized that the sample containing 0.5% TiO_2_ NPs differs in its melting peak shape, demonstrating a more pronounced low-temperature region. The second crystallization profile is identical to the first crystallization cycle for all the samples (Figure 4D): the onset of crystallization occurs at approximately 130–127 °C, and the process concludes at around 110 °C.

PVDF is a semicrystalline polymer that exists in several crystalline modifications.

When heated above 160 °C, metastable phases can transform into each other as a result of recrystallization under controlled conditions, so a single broad peak or “shoulder” is often visible on the DSC curve [36]. In the first heating, all thermograms have one complex melting peak with a maximum in the region of 155–158 °C. In Table 3, adding nanoparticles to the polymer matrix decreases the melting temperature and enthalpy of the material, which may be associated with the limitation of lamella growth caused by nanoparticles. It is important to emphasize that the changes are quite small and slightly beyond the margin of error. However, given that DSC samples are taken as a sample of 5 points, it is safe to say that structural differences correlate with the results given below. However, during the second heating, the separation of polymorphic modifications caused by the first melting is clearly visible (based on the appearance of the low- and high-temperature “shoulders” in the thermograms).

### 2.4. Contact Angle of Composite Films P(VDF/TFE)/TiO_2_NPs

The contact angle of polymer films can be used to compare the hydrophilicity and hydrophobicity of a material’s structure. Surface free energy is directly related to chemical composition, so the presence of more hydrophilic objects (molecules, particles, and groups) in the polymer matrix decreases the contact angle and vice versa. As can be seen from Table 4, the native polymer exhibits pronounced hydrophobic properties with a contact angle of approximately 104°, and adding 0.5% TiO_2_NPs does not change this value. However, adding 1% TiO_2_NPs reduces the contact angle to approximately 100°, demonstrating an increase in the material’s hydrophilicity.

### 2.5. Surface Morphology of Composite Films P(VDF/TFE)/TiO_2_NPs

**Figure 5:**
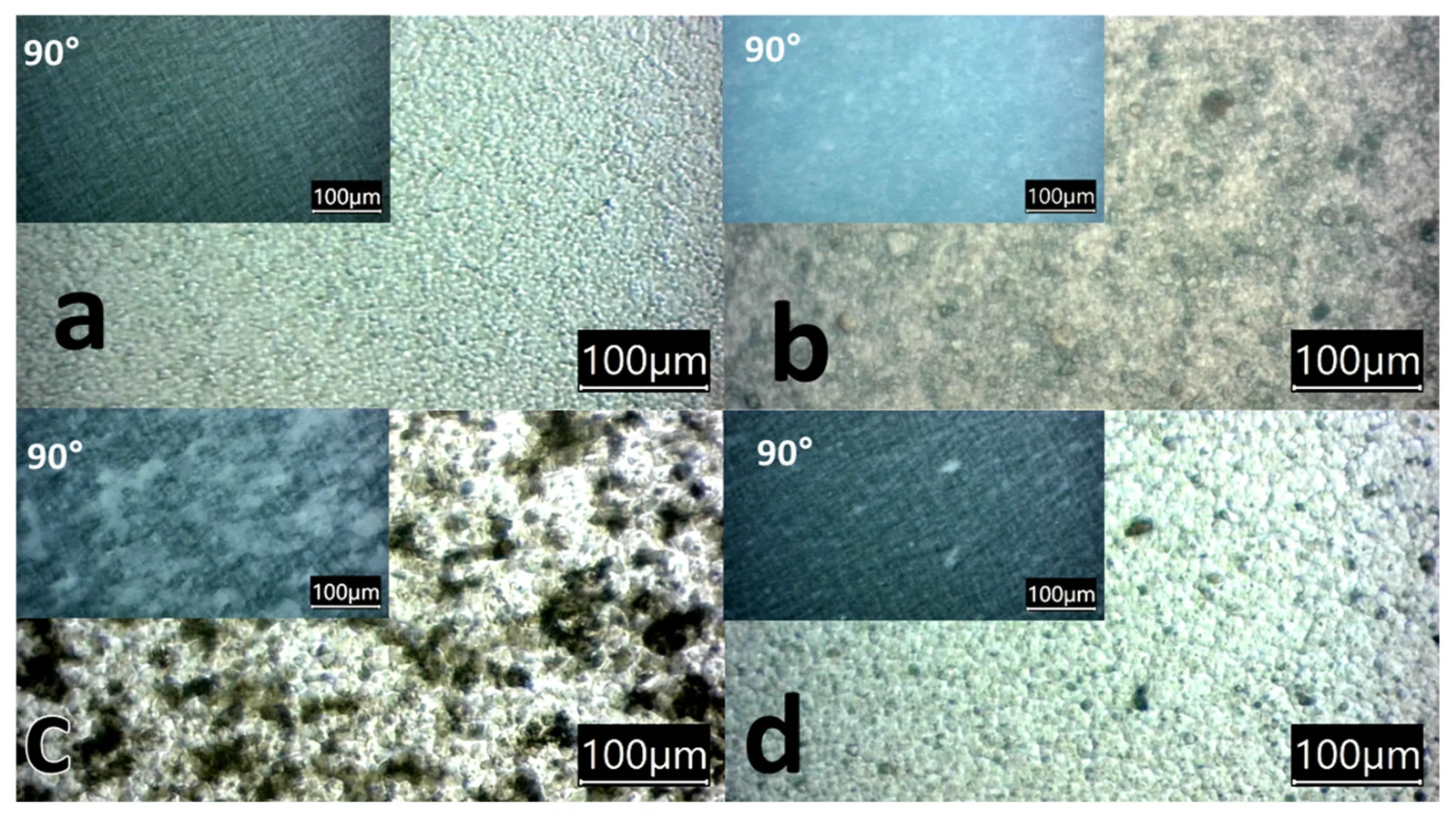
Optical micrographs of films with different TiO_2_NPs content: reference film (**a**), 5% (**b**), 1% (**c**), 0.5% (**d**), film image in crossed polarizers (inset).

The introduction of nanoparticles into the polymer matrix significantly alters the structural and morphological properties of the material. Figure 5 shows optical micrographs of the film surface with varying TiO_2_NPs content. To study optical anisotropy, the micrographs were taken in normal and polarized light (the angle between the polarizer and the analyzer was 90°).

Optical micrographs taken at a 0° viewing angle show that the films exhibit a spherulitic structure. The size of the spherulites on the film surface varies depending on the dopant concentration. The maximum spherulite size (7 µm) was demonstrated by the sample with 0.5% nanoparticles, while the minimum size (2 µm) was characteristic of the sample with 5% TiO_2_NPs.

In crossed polarizers (90° angle), the picture is significantly different. For the reference film, outlines resembling the classical 4-petal figure of α-spherulites are observed, but for all composite samples, such a picture is practically absent. According to the literature, ring spherulites with a characteristic 4-petal figure are mainly formed by the α-phase of P(VDF/TFE) [37], while ringless spherulites are characteristic of the γ-phase [38], which has a significantly lower birefringence. Thus, the suppression of the 4-petal pattern upon the introduction of TiO_2_ nanoparticles indicates the suppression of the α-phase in favor of the formation of polar phase crystallites. This conclusion correlates with IR spectroscopy data demonstrating an increase in the proportion of the β-phase from 37% for the reference film to 50% for the samples with TiO_2_NPs.

The interference color of the films in polarized light differs significantly (Figure 5, inset). Since the thickness of all samples is comparable (30 μm), it can be concluded that the color change indicates differences in the birefringence of the films. The difference in birefringence is due to changes in both phase composition and supramolecular structure. This is consistent with the assumption that the introduction of nanoparticles leads to structural rearrangement at the level of both the crystal lattice and the supramolecular structure of the polymer.

### 2.6. Transparency of Composite Films P(VDF/TFE)/TiO_2_NPs

**Figure 6:**
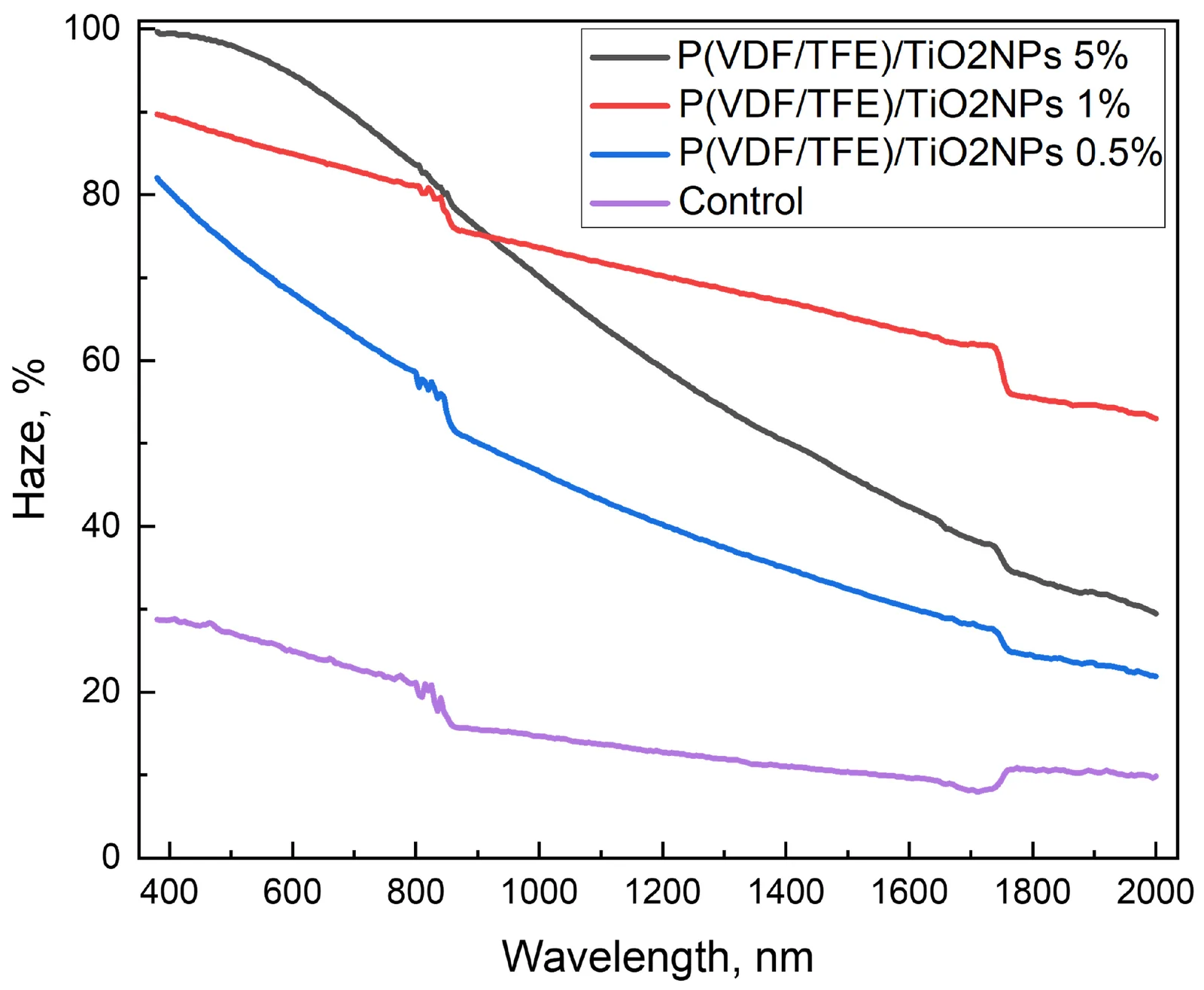
Visible and near FTIR spectroscopy of P(VDF/TFE)/TiO_2_NPs composite polymer films.

The transparency of a composite piezoelectric polymer material significantly impacts its integration into various sensors and wearable electronics. Figure 6 shows the dependence of haze on wavelength for P(VDF/TFE) composite film samples in the visible and near-infrared ranges. As can be seen from Figure 6, increasing the concentration of titanium dioxide nanoparticles in the polymer matrix significantly reduces transparency in the visible range and may limit the use of composite materials. Haze exhibits a concentration dependence on particle content in the polymer matrix. Samples with even a 0.5% content have a haze of over 60%. In the near-infrared range, haze decreases for samples with 0.5 and 1% content, and the difference between the pure film and samples containing particles is 10–17%. The transparency of piezoelectric films based on P(VDF/TFE) with titanium nanoparticles in the IR range will enable their comprehensive use in optoelectronic devices, sensors, and flexible photonic systems without significant attenuation of transmitted radiation.

### 2.7. Atomic Force Microscopy of P(VDF/TFE)/TiO_2_NPs

**Figure 7:**
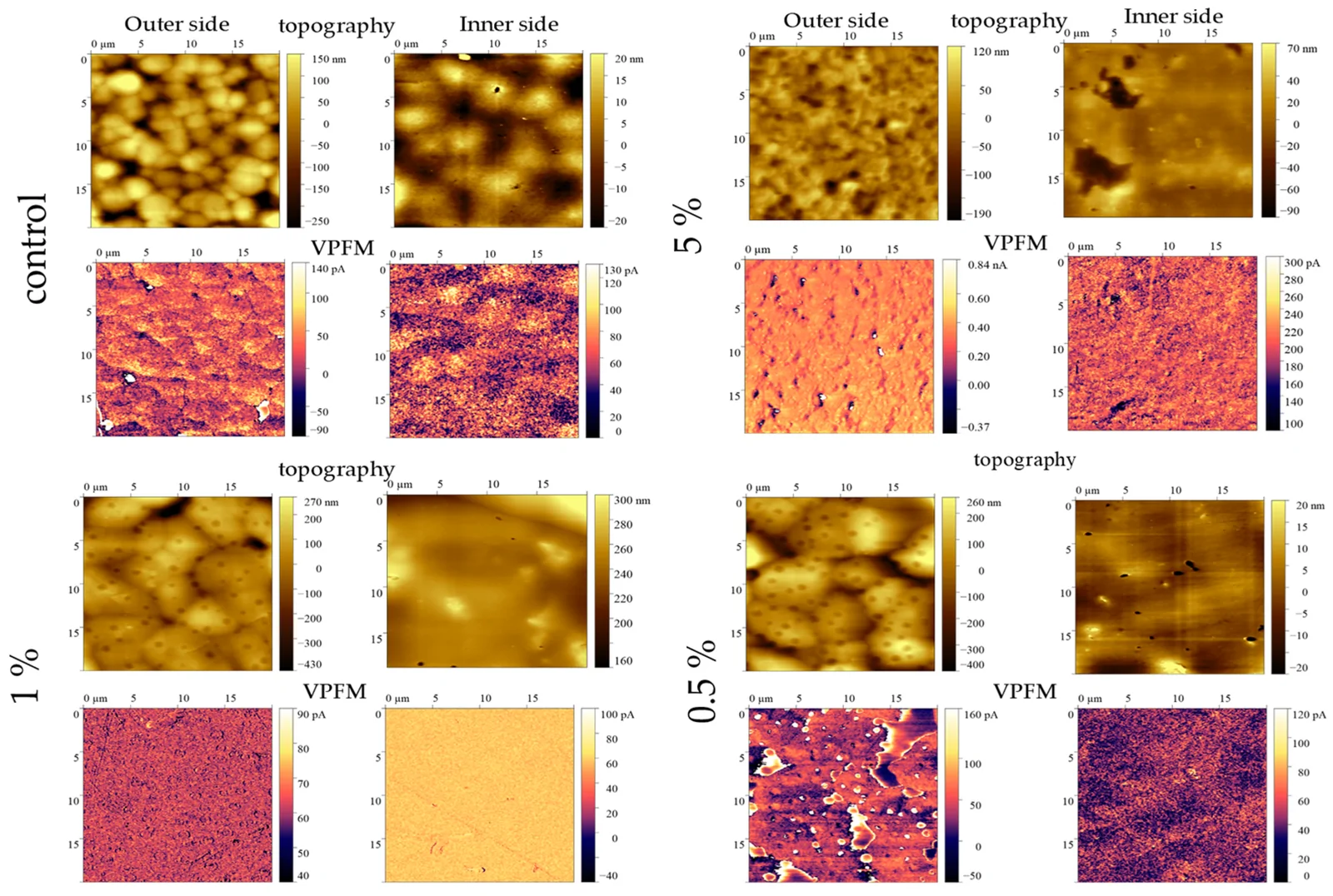
PFM data for films with different TiO_2_NPs content.

Figure 7 and Table 5 present Atomic Force Microscopy (AFM) data in piezoresponse force microscopy (PFM) mode for P(VDF/TFE) films with varying TiO_2_NPs contents.

As can be seen from the AFM data (Figure 5), surface morphology directly depends on the concentration of doped particles. The size of spherulites on the outer surface of the films (d) increases from 3 μm for the reference sample to 7 μm for the composite with 0.5% TiO_2_NPs, indicating the role of nanoparticles as effective crystallization centers facilitating the growth of large supramolecular structures. However, at 5% TiO_2_NPs, the spherulite size decreases to 2 μm. This may be due to the fact that an excess of nucleation centers and, possibly, particle agglomeration hinder the growth of large spherulites, forming a finer-grained structure (Table 6).

All samples in the series are characterized by non-zero vertical piezoelectric response signals in the uninduced state, which indicates the presence of spontaneous polarization.

PFM data confirm the ferroelectric nature of the composite films and suggest the possibility of further polarization to enhance the material’s overall dipole moment by reorienting and ordering ferroelectric domains under the influence of an external electric field.

### 2.8. Electrophysical Properties of Composite Films P(VDF/TFE)/TiO_2_NPs

**Figure 8:**
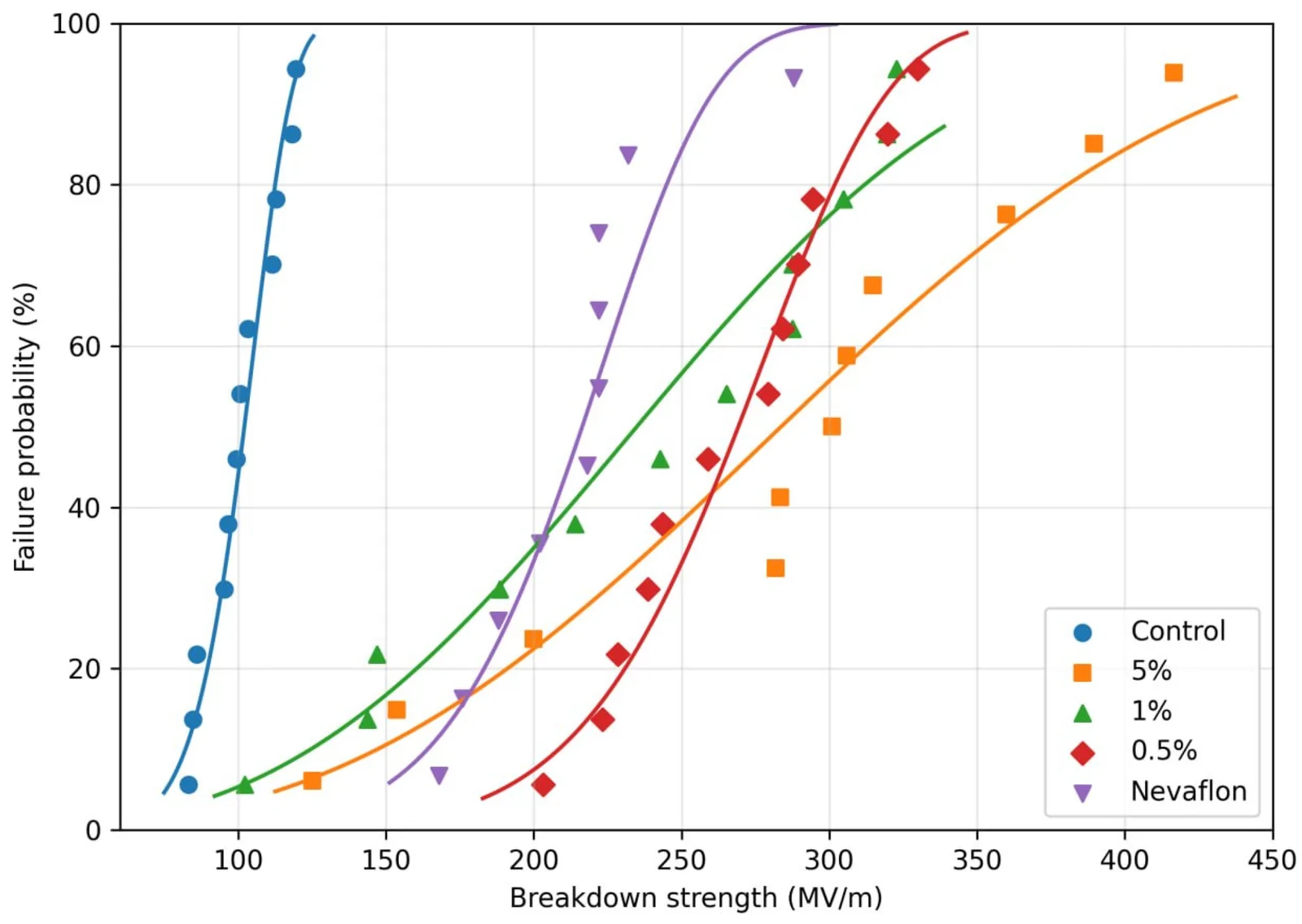
Breakdown characteristics of samples in Weibull coordinates.

The test samples were subjected to contact polarization at an electric field strength of 150 MV/m (Figure 8). Contact polarization of the films was performed without prior high-temperature orientational drawing. Table 5 presents the results of measurements of the electrophysical properties of the test samples.

As can be seen from the obtained data (Table 5), the introduction of TiO_2_NPs leads to the emergence of a piezoelectric response in the composites. Moreover, the maximum piezoelectric response value of 6.4 pC/N is achieved at a dopant concentration of 0.5 wt%. A relatively high piezoelectric response is also observed for a sample containing 1% TiO_2_NPs. However, a further increase in the TiO_2_NPs concentration to 5% decreases the piezoelectric properties of the material.

The introduction of nanoparticles into the polymer matrix increases the electric strength of the composite films from 110 to 320 MV/m. The electric strength of the composite films is higher than that of a commercially available P(VDF/TFE) film (210 MV/m). Figure 6 shows the breakdown characteristics of the samples in Weibull coordinates.

As shown previously (Table 2), the introduction of TiO_2_NPs does not significantly affect the phase composition of the polymer. Therefore, the observed increase in electrical strength cannot be explained by an increase in the proportion of the electroactive phase of the polymer. At the same time, according to the literature, the introduction of nanoparticles, even in small concentrations, promotes the formation of a more uniform and ordered supramolecular structure of the polymer, as well as a decrease in the average crystallite size [39]. The work [40] shows that the homogeneity of the supramolecular structure and a decrease in crystallite size have a more significant positive effect on electrical strength than a change in phase composition. The formation of smaller, uniformly distributed crystallites decreases structural defects, a decrease in stress concentration and, as a consequence, an increase in the breakdown resistance of the material. Thus, it can be assumed that the main contribution to the increase in the electrical strength of the composites is not so much the change in phase composition but rather the structural ordering of the polymer matrix induced by the presence of nanoparticles. Furthermore, the increase in the electrical strength of the composite films may be due to the fact that amorphous TiO_2_NPs act as charge traps, hindering the development of breakdown channels.

**Figure 9:**
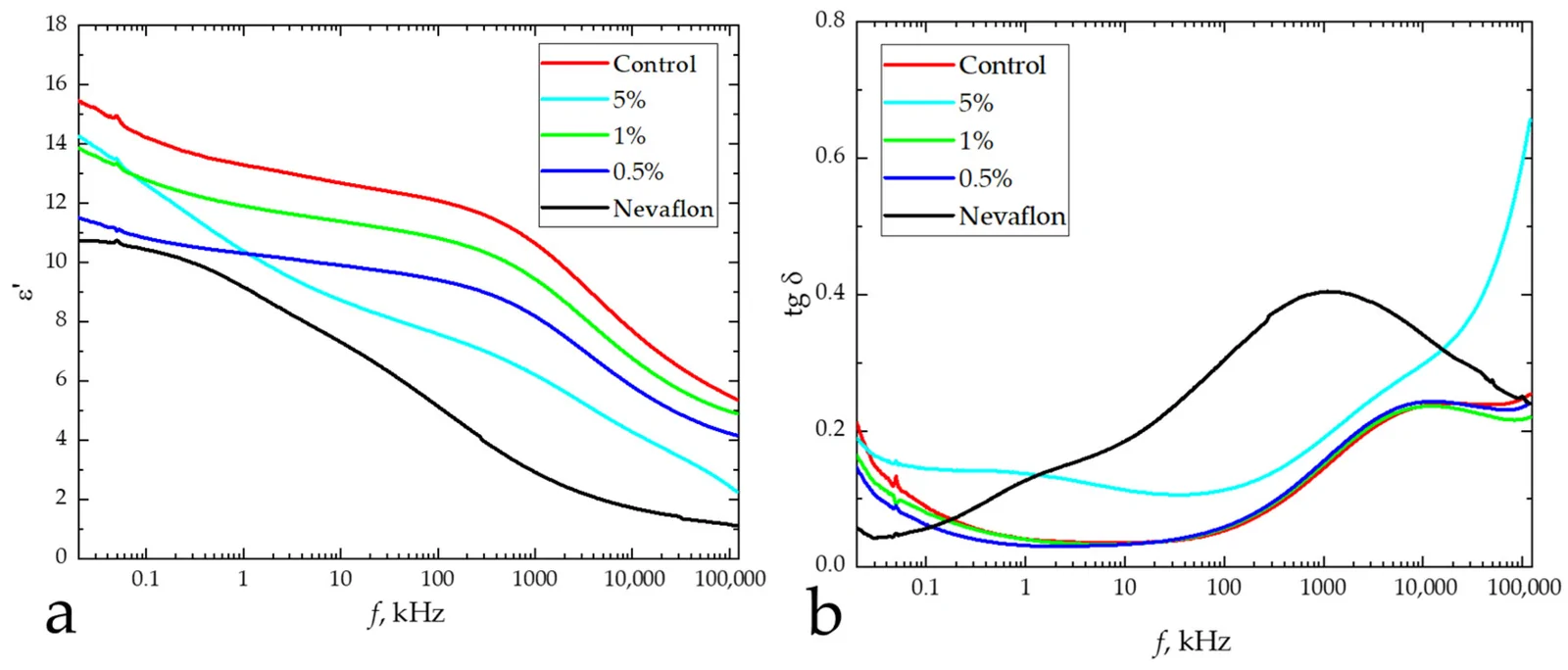
Frequency dependence of the real part of the dielectric constant (**a**) and the tangent of the dielectric loss angle (**b**) for pure and composite films.

The permittivity exhibits a non-monotonic dependence on concentration. An increase is observed at 0.5 wt.% (ε′ = 10.3), but at 1 and 5 wt.%, the ε′ values decrease to 10.4 and 11.9, respectively (Figure 9a).

The addition of nanoparticles into the polymer matrix leads to a decrease in the dielectric permittivity ε′ (from 13.9 for the control film to 10.3–11.9 for the composites). The most significant decrease in ε′ is observed for samples with 0.5 and 1 wt.%, which simultaneously exhibit the maximum values of the piezoelectric coefficient and electrical strength. This result can be explained in terms of the competition between two mechanisms. It is known that the manifestation of the piezoelectric effect in PVDF and its copolymers requires effective polarization, which is determined by the dielectric strength of the material. As shown above (Table 5, Figure 8), the introduction of nanoparticles leads to an increase in dielectric strength from 110 MV/m (control) to 290–320 MV/m for the composites. For the control film, the dielectric strength value (110 MV/m) is below the coercive field required for effective polarization and domain reorientation. The introduction of TiO_2_NPs increases the dielectric strength, enabling polarization and the emergence of a piezoelectric response.

The decrease in ε′ upon the introduction of nanoparticles can be associated with the restricted mobility of dipoles and segments of polymer chains near the particle surface, which reduces the contribution of dipolar polarization to the overall dielectric permittivity. At the same time, the dielectric loss tangent (Figure 9b) shows minimum values for the sample with 0.5 wt.% (tan δ = 0.031), which is consistent with its high electrophysical characteristics. The increase in losses at 5 wt.% (tan δ = 0.137) indicates enhanced relaxation processes at the polymer–nanoparticle interface, likely associated with particle agglomeration. It is important to note that the electrophysical characteristics of the obtained composite films (especially the 0.5 wt.% sample) significantly exceed those of the commercially available analogue (Nevaflon), confirming the promise of the developed approach.

## 3. Materials and Methods

### 3.1. Materials

A copolymer of vinylidene fluoride (94%) and tetrafluoroethylene (6%) was purchased from Halopolymer, Saint Petersburg, Russia; Titan(IV)-butoxid (TTIB) (CAS-244112), 2-Propanol (CAS-109634), and *N*,*N*-Dimethylformamide (CAS-227056) were purchased from Merck. A commercially available extruded film of a P(VDF/TFE) copolymer of 96/4 composition, manufactured by JSC Russian Research Center of Applied Chemistry (GIPH), was used as a reference sample for electrophysical measurements. The film thickness was 50 μm.

### 3.2. Synthesis of Titanium Dioxide Nanoparticles

Titanium butoxide (TTIB) (29.70 g) was dissolved in 6.23 g of isopropyl alcohol (IPA). Then, 5.98 g of this mixture was added dropwise over 3 min to 18.72 mL of water. The mixture was then stirred at 500 rpm and 50 °C for 3 h. The contents of the beaker were then poured into a Petri dish and dried in an oven at 80 °C for 3 h, followed by an additional calcination at 350 °C for 3 h.

### 3.3. Synthesis of P(VDF/TFE)/TiO_2_NPs Composite Film

Pre-synthesized TiO_2_NPs were suspended in *N*,*N*-Dimethylformamide (DMF) by ultrasonic treatment and homogenization. After that, a 5% solution of P(VDF/TFE) in DMF was mixed with a suspension of TiO_2_NPs (at the required concentration relative to P(VDF/TFE)) using ultrasonic treatment (30 min). The mixture was then stirred in a multirotator for 12 h, after which the mixture was poured into a Petri dish and kept in a drying oven for 5 h at 80 °C [41]. The thickness of the obtained films was ~30 µm.

As part of the study, composite films were obtained with the following dopant contents:

5% TiO_2_ nanoparticles;

1% TiO_2_ nanoparticles;

0.5% TiO_2_ nanoparticles;

Control—for comparison, a control sample was prepared without titanium dioxide nanoparticles but using the same method.

**Figure 10:**
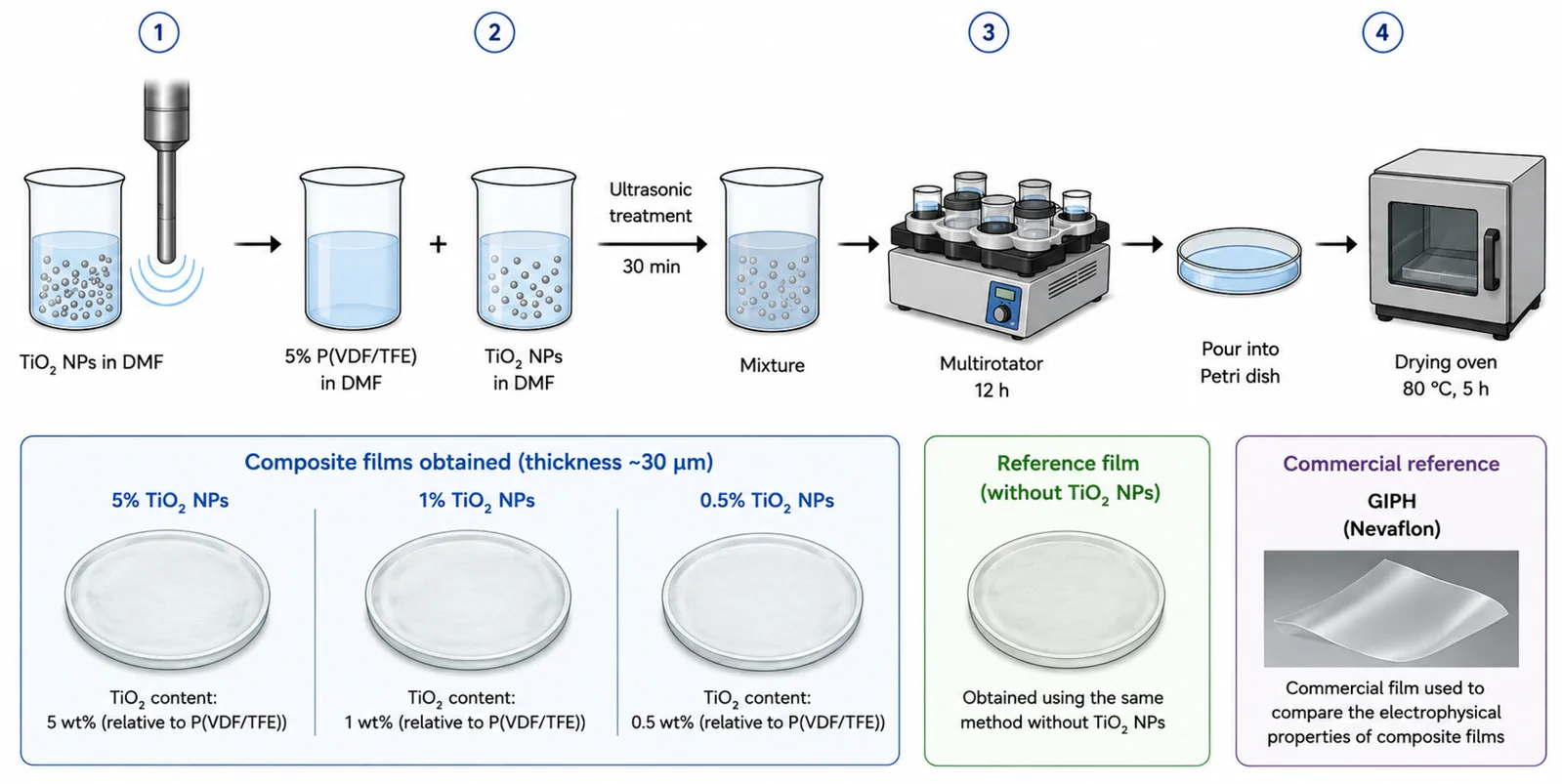
Scheme for obtaining P(VDF/TFE)/TiO_2_NPs films.

A reference film was obtained using the same method but without TiO_2_ nanoparticles. GIPH (Nevaflon) is a commercial film used to compare the electrophysical properties of composite films (Figure 10).

### 3.4. Characterization Methods

#### 3.4.1. X-Ray Diffraction Analysis

X-ray diffraction patterns of TiO_2_NPs were obtained using an X’Pert Pro MPD diffractometer (PANalytical, Almelo, The Netherlands) with a PIXcel high-speed detector. XRD was performed advance spectrometer with 1.541 A(Cu-Kα), with 2θ angles in the range from 0° to 85° and a scanning step Δ2θ = 0.05°. Cu Kα-radiation was used, which was further decomposed into Kα1 and Kα2 components during spectral processing.

#### 3.4.2. Dynamic Light Scattering (DLS) and Electrophoretic Light Scattering (ELS)

The hydrodynamic diameters of the synthesized nanoparticles, as well as their zeta potentials in water, were determined using a Zetasizer Nano S ZEN3600 (Malvern Instruments, Worcestershire, UK) with a 633 nm helium-neon laser light source and a maximum power of 4 mW (scattering angle of 173°).

Measurements were performed in special quartz cells at 25 °C. The results presented are averaged over three independent measurements. The nanoparticle suspension was not subjected to any additional processing after homogenization in water.

#### 3.4.3. FTIR Spectroscopy

Fourier-transform infrared (FTIR) spectrometry of composite film samples was performed using an FT-803 spectrometer (Simex, Novosibirsk, Russia) with an attenuated total reflectance (ATR) accessory (diamond crystal). The optical density of the samples was recorded in the wavenumber range of 550–4000 cm^−1^. Spectral processing consisted of spectral smoothing (using nine points) and baseline subtraction (conversion to the required units of measurement, cropping, etc.) using OPUS7.2 software (Bruker GmbH, Moscow, Russia).

The film phase composition (electroactive phase fraction and α/am-phase relation) was computed using transmission FTIR spectroscopy based on the literature data [36].

Electroactive phase fraction:

(1) $$F_{ea}=\frac{A_{837}}{1.26\cdot A_{763}+A_{837}},$$

where *A*_763_ and *A*_837_ are the amplitudes of peaks centered at 763 and 837 cm^−1^ (*α*-phase and electroactive (*β* and *γ*) phase peaks), respectively.

1.26—This is a coefficient that takes into account the difference in extinction (absorption) coefficients of the *α*- and *β*-phases of PVDF when using bands around 763 and 837 cm^−1^.

#### 3.4.4. Differential Scanning Calorimetry (DSC)

Differential Scanning Calorimetry (DSC) measurements were carried out using a Netzsch 214 Polyma (Netzsch, Selb, Germany) in a Nitrogen atmosphere. The heating and cooling rate was 10 °K/min. The PVDF samples were cut from 3 different areas of the films with a total weight of 2–5 mg. The enthalpy of melting (∆H_melting_), melting temperature (T_melting_), the enthalpy of crystallization (∆H_crystallization_), and crystallization temperature (T_crystallization_) were calculated using Netzsch Proteus software, version 9.9.

The degree of crystallinity was calculated from the enthalpy of melting using Formula (2):

(2) $$\chi_{c}\;=\;\frac{∆H_{m}}{\left(a\cdot ∆H_{m}^{\alpha }+b\cdot ∆H_{m}^{\mathrm{ea}}\right)},$$

where: ∆*H_m_* is the film melting enthalpy; $∆H_{m}^{\alpha }$ is the melting enthalpy of the *α*-phase, which is 93.07 J/g; $∆H_{m}^{\mathrm{ea}}$ is the melting enthalpy of the electroactive phases (equal for *β*- and *γ*-phases), which is equal to 103.4 J/g [42].

#### 3.4.5. Polarization Microscopy

Using an A15.1302 Polarizing Microscope (Opto-Edu, Beijing, China), we obtained micrographs of the film surfaces and studied the effect of TiO_2_NPs concentration on the polymer’s supramolecular structure and optical properties. Imaging was performed at two polarizer–analyzer configurations (0° and 90°): the former yielded conventional bright-field images of the surface morphology, while the latter allowed visualization of the two-dimensional scattering indicatrix, providing insights into the structural ordering of the material.

#### 3.4.6. VIS-Spectroscopy

The optical transmittance (*T*(*λ*)) of the samples was measured in the wavelength range from 380 to 780 nm using a UV-3600i Plus dual-beam spectrophotometer (Shimadzu, Kyoto, Japan). Two measurement configurations were employed: in free space with an 8 nm slit width and with an integrating sphere with a 32 nm slit width. The spectral resolution of the instrument was 5 nm in all experiments. The total transmittance (τt) for the CIE standard illuminant D65 was calculated according to the method described in ISO 13468-2:2021 [43].

Haze (*H*(*λ*)) was calculated using Formula (1) in accordance with the method described in ISO 14782:2021 [44]:

(3) $$H\left(\lambda \right)=\frac{Tsphere\left(\lambda \right)-Tdirect(\lambda )}{Tsphere(\lambda )},$$

where *Tsphere*(*λ*) is the transmittance coefficient measured with the integrating sphere; *Tdirect*(*λ*) is the transmittance coefficient measured in the free-space configuration; *λ* is the wavelength (nm).

#### 3.4.7. Contact Angle

Contact angle measurements were performed using a DSA25 Drop Shape Analyzer (Krüss, Hamburg, Germany). All measurements were conducted at temperatures of 20–25 °C with a 3 µL drop of solvent applied to the sample. Each measurement represented the average contact angle from five measurements.

### 3.5. Electrophysical Properties

Electric strength measurements were performed in a bath filled with Vaseline oil. Ten measurements were recorded for each sample. Breakdown field strength values were determined using the 2-parameter Weibull model [45,46], according to which a statistical set of a large number of field values Eb can be described by the function

(4) $$F\left(x\right)=1\;-\;\exp[-{\left(\frac{x}{\alpha }\right)}^{\beta_{b}}],$$

where x is the current value of Eb, α is a certain characteristic field at which at least 63.2% of the test samples undergo breakdown; the parameter β_b_ characterizes the variance of Eb relative to the mean.

The dielectric properties of the materials were studied using broadband impedance spectroscopy with an E7-28 Immittance Analyzer (MNIPI, Minsk, Belarus) in the frequency range from 25 Hz to 10 MHz. Prior to measurements, copper electrodes 200 nm thick and 5 mm in diameter were applied to the samples.

Macroscopic piezoelectric coefficients d_33_ were determined by the quasi-static Berlincourt method on a d33-meter YE2730A (Sinocera Piezotronics, INC, Yangzhou, China).

Local piezoelectric properties and domain structure changes were studied using scanning probe microscopy on an NTEGRA Prima microscope (NT-MDT SI, Zelenograd, Russia) with FMG01/Pt platinum-coated conductive cantilevers (Tipsnano, Tallinn, Estonia). Experimental data were processed using Gwiddion 2.69 software (Czech Metrology Institute, Brno, Czech Republic). The characteristic domain size ξ was found by calculating the correlation function of the vertical piezoresponse signal in relation to the coordinate r:

(5) Cr = A·exp−rξ2h,

where A is a constant, r is the distance from the central peak (nm) determined from the autocorrelation function image, ξ is the average domain size (nm), and h (0 < h < 1) is a parameter [47].

## 4. Conclusions

This study developed a method for producing composite films based on a P(VDF/TFE) copolymer modified with titanium dioxide nanoparticles (TiO_2_NPs) using casting technology. A comprehensive study was conducted to examine the effect of the concentration of introduced nanoparticles on the structural characteristics, phase composition, and surface morphology of the resulting composite materials. It was found that the addition of as little as 0.5 wt% TiO_2_NPs leads to significant changes in the degree of crystallinity and phase composition of the P(VDF/TFE) copolymer, indicating a pronounced influence of the nanoparticles on the formation of the polymer structure. Atomic force microscopy and contact angle analysis showed that increasing the TiO_2_NPs content to 1 wt% is accompanied by a noticeable change in surface microrelief, increased roughness, and modification of the surface properties of the composite material, which may be related to specific features of film surface structure formation.

It was shown that the introduction of TiO_2_NPs into a VDF/TFE polymer matrix increases the electric strength of composite films by more than 2.5 times (up to 320 MV/m versus 110 MV/m for the pure polymer). Doping with NPs facilitates efficient polarization of the material without preliminary high-temperature orientational drawing. The piezoelectric coefficient d_33_ of the composite film is 6.4 pC/N. A correlation has been established between electrical strength and the possibility of polarization: an increase in Eb with the introduction of TIO_2_NPs to values exceeding the coercive field ensures the effective formation of a piezoelectric response. At the same time, a decrease in the dielectric constant of composites is associated with a limitation of the mobility of dipoles and can be considered as evidence of the structural ordering of the polymer matrix.

These results demonstrate the potential of the developed composites for use in flexible piezoelectric devices, optical sensors, and biomedical applications.

## Figures and Tables

**Table 1:** Performance of titanium dioxide nanoparticles obtained using DLS and ELS.

Characteristics	Z-Average (d. nm)	Zeta Potential (mV)	St. Dev. (Series)	PDI
TiO_2_NPs	215.7	24.2	1.747	0.177

**Table 2:** Calculation of the phase composition of the polymer included in the P(VDF/TFE)/TiO_2_NPs composite, based on IR spectroscopy data and the degree of crystallinity (χ) from DSC data.

Sample	$A_{763}^{\alpha }$	$A_{837}^{EA}$	$A_{1234}^{\gamma }$	$A_{1275}^{\beta }$	EA-Phase, %	β-Phase, %	χ, %
Control	0.37	1.63	0.94	0.56	77.9	37.2	32.2
P(VDF/TFE)/TiO_2_NPs 0.5%	0.38	1.61	0.72	0.68	76.9	48.9	32.2
P(VDF/TFE)/TiO_2_NPs 1%	0.36	1.59	0.70	0.72	77.6	50.8	28.4
P(VDF/TFE)/TiO_2_NPs 5%	0.32	1.38	0.78	0.78	77.1	50.0	30.1

**Table 3:** Thermal properties of the PVDF samples.

**Sample**		**1 Cycle**			
		**T_melting_ (°C)**	**∆H_melting_ (J/g)**	**T_crystallization_ (°C)**	**∆H_crystallization_ (J/g)**
5%		157 ± 2	32 ± 3	120 ± 2	−26 ± 3
1%		156 ± 2	30 ± 3	120 ± 2	−23 ± 3
0.5%		155 ± 2	34 ± 3	120 ± 2	−29 ± 3
Control		158 ± 2	35 ± 4	119 ± 2	−30 ± 4
**Sample**	**2 Cycle**				
	**T_melting_ (°C)**		**∆H_melting_ (J/g)**	**T_crystallization_ (°C)**	**∆H_crystallization_ (J/g)**
	**1st Peak**	**2nd Peak**			
5%	149 ± 2	159 ± 2	29 ± 3	120 ± 2	29 ± 3
1%	148 ± 2	159 ± 2	25 ± 3	119 ± 2	33 ± 3
0.5%	148 ± 2	157 ± 2	22 ± 2	119 ± 2	31 ± 3
Control	147 ± 2	156 ± 2	22 ± 2	120 ± 2	32 ± 3

**Table 4:** Dependence of the composite’s contact angle on the concentration of doped TiO_2_NPs.

Sample	Control	0.5%	1%	5%
Average contact angle, °	103.8 ± 3.55	108.98 ± 3.43	100.83 ± 1.4	99.07 ± 3.25
Droplet images	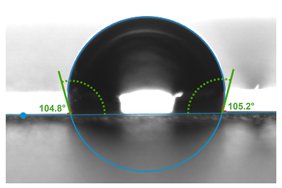	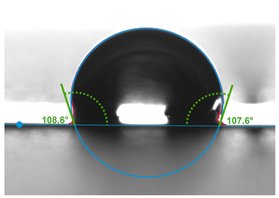	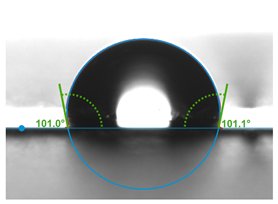	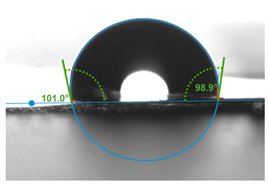

**Table 5:** Electrophysical properties of composite films.

Sample	d_33_, pC/N	Eb, MV/m	ε′ (1 kHz)	tg δ (1 kHz)
Control	0	110	13.9	0.040
5%	4.5	320	10.4	0.137
1%	6.3	270	11.9	0.040
0.5%	6.4	290	10.3	0.031
Nevaflon	5.0	210	9.2	0.127

**Table 6:** Scanning probe microscopy data.

Sample	Reference		5%		1%		0.5%	
Side	Inner	Outer	Inner	Outer	Inner	Outer	Inner	Outer
RMS, nm	9	84	18	35	16	87	6	112
*d*, μm	4	3	–	2	–	6	–	7
ξ, nm	98	204	138	215	27	201	35	72

## Data Availability

The original contributions presented in this study are included in the article. Further inquiries can be directed to the corresponding author.
